# Optimization of the Synthesis of Natural Polymeric Nanoparticles of Inulin Loaded with Quercetin: Characterization and Cytotoxicity Effect

**DOI:** 10.3390/pharmaceutics14050888

**Published:** 2022-04-19

**Authors:** Jocelyn C. Ayala-Fuentes, Melissa Zulahi Gallegos-Granados, Luis Jesús Villarreal-Gómez, Marilena Antunes-Ricardo, Daniel Grande, Rocio Alejandra Chavez-Santoscoy

**Affiliations:** 1 Tecnológico de Monterrey, Escuela de Ingeniería y Ciencias, Ave. Eugenio Garza Sada 2501 Sur, Monterrey 64849, Mexico; a01329401@tec.mx; 2 Facultad de Ciencias Químicas e Ingeniería, Universidad Autónoma de Baja California, Tijuana 22260, Mexico; zulahi.gallegos@uabc.edu.mx (M.Z.G.-G.); luis.villarreal@uabc.edu.mx (L.J.V.-G.); 3 Facultad de Ciencias de la Ingeniería y Tecnología, Universidad Autónoma de Baja California, Tijuana 22260, Mexico; 4 Tecnologico de Monterrey, The Institute for Obesity Research, Ave. Eugenio Garza Sada 2501 Sur, Monterrey 64849, Mexico; marilena.antunes@tec.mx; 5 Univ Est Creteil, CNRS, Institut de Chimie et des Matériaux Paris-Est (ICMPE), 2, rue Henri Du-nant, 94320 Thiais, France; grande@icmpe.cnrs.fr

**Keywords:** inulin, flavonoid, nanoparticle, quercetin

## Abstract

Quercetin is a bioactive component that is capable of having therapeutic potential in the prevention of different noncommunicable chronic diseases (NCDs). However, it presents instability in the gastrointestinal tract in addition to low bioavailability. One way to overcome the limitations of quercetin lies in using nanotechnology for the development of nanoparticles, based on biopolymers, that are capable of being ingestible. Inulin, a fructan-type polysaccharide, acts as a delivery system for the release of quercetin in a target cell, guaranteeing the stability of the molecule. Inulin-coated quercetin nanoparticles were synthesized by the spray dryer method, and four variables were evaluated, namely inulin concentration (5–10% *w*/*v*), feed temperature (40–60 °C), inlet temperature (100–200 °C) and outlet temperature (60–100 °C). The optimal conditions were obtained at 10% *w*/*v* inulin concentration, with 45 °C feed temperature, 120 °C inlet temperature and 60 °C outlet temperature, and the nanoparticle size was 289.75 ± 16.3 nm in water. Fluorescence microscopy indicated quercetin loading in the inulin nanoparticles, with an encapsulation efficiency of approximately 73.33 ± 7.86%. Inulin-coated quercetin nanoparticles presented effects of inhibition in Caco-2 and HepG2 cells, but not in HDFa cells. The experimental data showed the potential of inulin nanoparticles as transport materials for unstable molecules, in oral administration systems, for the encapsulation, protection and release of quercetin.

## 1. Introduction

In the last 25 years, noncommunicable chronic diseases (NCDs) have been one of the greatest challenges faced by the health sector due to the increase in mortality around the world [1]. NCDs constitute a group of conditions that result in long-term health consequences and require long-term treatment and care. These conditions include cancers, cardiovascular disease, diabetes and chronic lung illnesses [2]. In addition, reports suggest that the prevention of NCDs by consuming vegetables and fruits is related to their contents of secondary metabolites, to which a beneficial bioactivity for health is attributed [3,4]. Moreover, due to their properties, these secondary metabolites represent an important impact on the global economy. According to a British Broadcasting Corporation (BBC) report, the global market for plant-derived bioactive compounds will grow, with an average annual growth rate of 6.1% forecasted from 2017 until 2022 [5].

The chemical structure of flavonoids is composed of a variable number of hydroxyl groups, thus potentiating antioxidative, anti-inflammatory, anti-mutagenic and/or anti-carcinogenic properties. They are potential inhibitors of several enzymes, such as xanthine oxidase (XO), cyclo-oxygenase (COX), lipoxygenase and phosphoinositide 3-kinase [4,5,6]. Although flavonoids have health implications for humans, their bioavailability is generally low and can vary drastically among different flavonoid classes and among individual compounds within a particular class [7,8].

Quercetin is a flavonoid and natural antioxidant that is extracted from vegetables and fruits to treat many diseases. Quercetin was shown to exert beneficial health effects in various animal and human cellular models by modulating signaling pathways and the gene expression involved in these processes [9]. The intake of quercetin has a positive correlation with the promotion of health. It can be ingested as a supplement, with a recommended daily intake of between 200 and 1200 mg, or as a nutraceutical through functional foods, at quantities of between 10 and 125 mg per serving [10].

Most of the quercetin in plants (or quercetin-rich foods) is attached to sugar molecules rather than occurring in its free form, and presents as a conjugate known as glycoside. Its sizes and polarities can cause difficulties in the absorption process in the gut [11]. The relatively low bioavailability of quercetin is attributed to its low absorption, extensive metabolism and/or rapid elimination [12].

Bioactive compounds are difficult to incorporate into food because of their limited chemical stability. However, nanoencapsulation has the potential to enhance bioavailability because particle size has a significant effect on the delivery of compounds to various sites within the body; when the size decreases, the bioavailability increases [13]. In addition, nanoparticles have been demonstrated to increase solubility and to have the capacity to penetrate the blood–brain barrier (BBB), enter the pulmonary system and be absorbed through the right connections of endothelial cells in the skin [14,15,16]. One of the latest strategic therapeutic possibilities for the vectorization of drugs and the boosting of the safety and efficacy of therapies is the use of nanocomposites for drug delivery [17].

Controlled incorporation and release from the external polymeric membrane are vital in terms of nanocapsule synthesis for many pharmaceutical agents. The physicochemical properties of the selected polymer play a crucial role in the responsiveness of the nanomaterial. Inulin is a flexible, fructan-type polysaccharide carbohydrate, and is mainly obtained from the root of chicory. It is a water-soluble dietary fiber and has been recently approved by the Food and Drug Administration. Moreover, due to its not being digested or fermented in the initial portion of the human digestive system but instead directly reaching the distal portion of the colon, inulin has been categorized with Generally Regarded As Safe (GRAS) status [18,19]. Inulin is specifically degraded into the colon by different types of microbiota [20]. Moreover, inulin promotes the growth of bifidobacteria, bacteroides and lactobacilli [21]. As a result, inulin might serve as a matrix for nanoparticles, as well as a prebiotic and a carrier.

The major target issues and routes for exposure to oral nanoparticles are the intestine and the liver [22]. Nowadays, several in vivo studies show that nanoparticles accumulate in the liver, which plays the most important role in the cleaning of substances, including nanoparticles, as its natural function is to eliminate foreign substances [23]. One of the most common reasons for medicines to be rejected or for nanoparticles to be removed from the market is the induction of hepatotoxicity [24]. In addition, in some studies, to screen for cytotoxicity of novel compounds or carriers, nontumoral cell lines such as human dermal fibroblast (HDFa) are used [25].

Thus, this study aimed to design and optimize the synthesis of loaded quercetin (NQ) nanoparticles in an inulin-based polymeric matrix. The optimized formulation (NQs) was also characterized based on size, polydispersity index (PDI) and zeta potential. NQs were characterized regarding their physicochemical characteristics and stability using scanning electron microscopy (SEM), Thermogravimetric analysis (TGA) and X-ray diffraction (XRD). A mathematical model was used to describe NQs’ in vitro drug release. To assess the potential efficacy of NQs in NCD prevention, in vitro cell viability assays were conducted on a human colon cancer cell line (Caco-2), a human hepatic cancer cell line (HepG2) and a human dermal fibroblasts (HDFa) cell line. Finally, the anti-inflammatory potential of NQs was evaluated by means of a Nitric Oxide Assay. 

## 2. Materials and Methods

### 2.1. Materials and Chemicals

Organic inulin from Agave (6179) powder (Microingredients^®^, Montclair, CA, USA). In addition, quercetin 95% (HPLC, Sigma Aldrich, San Luis, MO, USA), Absolute ethanol 99.5% (Sigma Aldrich, San Luis, MO, USA), Milli Q (Milli-Q Integral 15 Equipment, Merck México, Estado de Mexico, Mexico), distilled water (Milli-Q Integral 15 Equipment, Merck México, Estado de Mexico, Mexico), Acetonitrile 99.95% (DEQ), Methanol 99.96% (J.T. Baker), Acetone 99.95% (Sigma Aldrich, San Luis, MO, USA), HCl acid 37.40% (Sigma Aldrich, San Luis, MO, USA), NaOH 99.95% (Sigma Aldrich, San Luis, MO, USA) and glacial acetic acid 99.96% (Sigma Aldrich, San Luis, MO, USA) were obtained.

### 2.2. Optimization of Spray-Drying Process

For the optimization of the nanoparticles (loaded and not loaded with quercetin), different experiments were conducted, presenting a face-centered central composite design (CCD) in the study of four main variables, which included the inulin concentration percentage, feed temperature (Feed Temp.), inlet temperatures (Temp. in) and outlet temperatures (Temp. out) of the spray dryer equipment (described in Table 1). The response variables were the size (nm), polydispersity index (PDI) and Z-Average. The response surface methodology (RSM) with 5 axial points was used to minimize the dependent variables [26].

The recovery performance of the spray drying was calculated using the following equation:

(1) $$\mathrm{Equipment}\;\mathrm{efficient}\;(\%)=\frac{\mathrm{Final}\;\mathrm{mass}\;\mathrm{recovery}\;}{\mathrm{Total}\;\mathrm{solid}\;\mathrm{in}\;\mathrm{initial}\;\mathrm{solution}}\;\times \;100$$

### 2.3. Synthesis of Inulin Nanoparticles

For the optimal production of inulin nanoparticles loaded with quercetin (NQs), a stock solution of 450 mg of quercetin in 5 mL of absolute ethanol was prepared and then it was titrated to obtain a 10% ethanol solution in water, which was subjected to sonication at 40 KHz frequency, at no more than 25 °C, for 30 min. This solution was added dropwise to an aqueous solution of inulin at 10% (*w*/*v*). It was left under magnetic stirring at a temperature of 45–50 °C for 30 min [27].

**Figure 1:**
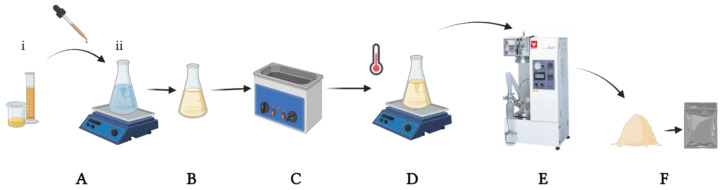
Experimental spray-drying process. (**A**) Quercetin solution (1:5 ethanol: water) (i) was added drop-by-drop to inulin solution (ii). (**B**) Mixed solution, inulin and quercetin homogenization. (**C**) Sonication at 25 °C for 30 min. (**D**) Final solution was agitated and maintained a specific temperature before and during drying. (**E**) Yamato model spray dryer. (**F**) NQs in a dust formulation were stored in dark conditions.

The preparation of inulin nanoparticles was performed using a laboratory-scale spray dryer (Yamato ADL-311, Santa Clara, CA, USA) with a nozzle setting of 0.406 mm. During the spray-drying process, all sample solutions were well diffused and thermostatised using a digital hot plate stirrer (VWR VMS-C7, Corning Mexicana, Nuevo Leon, Mexico). The feed rate was 3 mL/min, the inlet temperature was 120 °C and the outlet temperature was 60 ± 5 °C, with a pressure of between 0.15 and 0.2 MPa (Figure 1). The obtained powder was kept in dark hermetic bags (Whirl-Pak^®^, Merck Mexico, Ciudad de México, Mexico).

### 2.4. Characterization of NQs

#### 2.4.1. Dynamic Light Scattering (DLS)

The average size, and size distribution and polydispersity index (PDI) of the NQs and NBs were measured by dynamic light scattering (Zetasizer DTS 1060, Malvern instruments, Malvern, UK) and using the Zetasizer software (Malvern instruments, Malvern, UK). For the reconstruction of the samples of loaded and unloaded nanoparticles, 4 mg of NQs or NBs was diluted in 10 mL of 30% ethanol solution, and then the samples were sonicated for 10 min before reading at a refractive index of 1.450 and with dispersion optics at 173 °C (λ = 633 nm), in triplicate. The chemical stability of loaded (NQs) and unloaded (NBs) nanoparticles was also measured in the solution. The study was performed in triplicate.

#### 2.4.2. Scanning Electron Microscopy (SEM)

Scanning Electron Microscopy (SEM) was performed with a MERLIN microscope (Zeiss, Cambridge, UK) equipped with InLens, EBSD and SE2 detectors using a low accelerating tension (2–3 kV) with a diaphragm aperture of 30 μm. Prior to the analyses, the samples were coated with a 4-nm layer of palladium/platinum alloy in a Cressington 208 HR sputter-coater. Energy-dispersive X-ray spectroscopy (EDX) was performed using an SSD X-Max detector of 50 mm^2^ from Oxford Instruments (127 eV for the Kα ray of Mn).

#### 2.4.3. Fluorescence Microscopy (FM)

Fluorescence Microscopy (FM) measurements were carried out on an EVOSFLc inverted fluorescence microscope (Thermo Fisher Scientific, Waltham, MA, USA). Two fluorescence filters, DAPI (352–477 nm) and GFP (457–538 nm), were used, with an image magnification of 60×.

### 2.5. Zeta Potential

The surfaces of NB and NQ samples were detected using the Zetasizer DTS 1060 (Zetasizer DTS 1060, Malvern instruments, Malvern, UK) and the Zetasizer software (Zetasizer DTS 1060, Malvern instruments, Malvern, UK). For the reconstruction of the sample for loaded (NQs) and unloaded (NBs) nanoparticles, 4 mg quantities of NBs and NQs were diluted in 10 mL of 30% ethanol, then the samples were sonicated for 10 min before reading at 37 °C in triplicate. For chemical determination, the zeta potential was also determined in samples solubilized in an aqueous solution. 

### 2.6. Encapsulation Efficiency 

In order to determine the total amount of quercetin in NQs, and the amount of quercetin not encapsulated by the inulin matrix, the methodology proposed by Palma et al., [28] was used with slight modifications; these modifications are described in each of the following sections. Measurement was conducted in UV-vis (λ = 320 nm) in triplicate.

#### 2.6.1. Total Flavonoids

NQs (100 mg) were dispersed in 4 mL of water:ethanol:acetone (50:25:25 *v*/*v*/*v*), stirred for 1 min, passed to the ultrasonicator for 20 min at room temperature and centrifuged for 30 min at 3500 rpm. An aliquot of the supernatant was measured in UV-vis (λ = 320 nm). The study was performed in triplicate.

#### 2.6.2. Free Flavonoids

NQ s (100 mg) were placed in 4 mL of methanol. It was shaken very gently by hand for 2 min. The methanol helped to extract quercetin from the wall without breaking the polymer matrix. The supernatant was subjected to a water: methanol: acetonitrile (45:40:15 *v*/*v*/*v*) solution plus 1% glacial acetic acid. The supernatant was read in UV-vis (λ = 320 nm). The study was performed in triplicate.

#### 2.6.3. Encapsulation Efficiency (EE)

The encapsulation efficiency was compared with a predetermined calibration curve of quercetin (R^2^ = 0.96). The following formula was used [29].

EE (%) = Qa/Q_0_ × 100 (2)

where Qa represents the final quercetin (mg/mL) and Q_0_ (mg/mL) represents the initial quercetin.

### 2.7. Thermogravimetric Analysis (TGA) 

Thermogravimetric analysis (TGA) measurements were performed on a Setaram Setsys Evolution 16 thermobalance (Mettler-Toledo SAS, Virofla, France) by heating the samples at a rate of 10 °C min^−1^ from 20 °C to 800 °C under argon atmosphere. The assay was performed in triplicate.

### 2.8. Powder X-ray Diffraction (XRD)

X-ray diffraction (XRD) analyses were performed with a Bruker D8 DA VINCI diffractometer equipped with a Lynxeye rapid detector (Malvern Panalytical, Malvern, UK). The analysis conditions were set as follows: 2-thêta angle—5°–80°; scan mode—fast continuous PSD; time per step—0.3 s.

### 2.9. In Vitro Drug Release 

**Figure 2:**
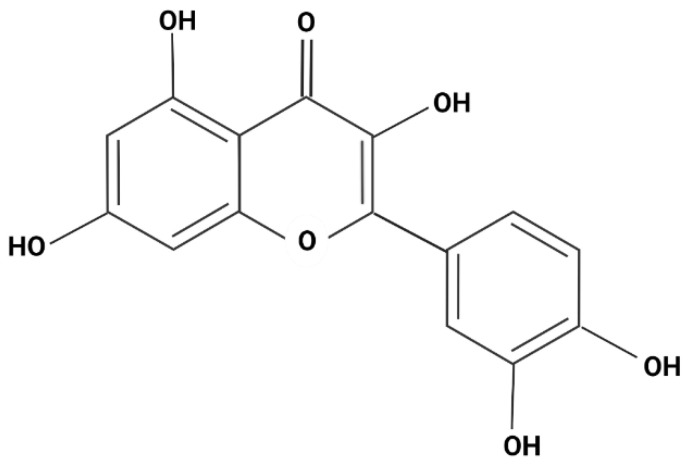
Molecular structure of quercetin.

The cumulative release of quercetin was calculated from a previously made standard curve. Figure 2 shows the molecular structure of quercetin, where an absorption scan was carried out with a known concentration of quercetin in ranges from 100 to 800 nm in UV-vis. This resulted in two peaks (λ1 = 318, λ2 = 574 nm).

Two calibration curves were formulated (pH 7.4, at 25 °C, R^2^ = 0.9911; pH 7.4, at 37 °C, R^2^ = 0.993). All experiments were performed in triplicate.

For the quantification of free quercetin as a function of time, 1.5 mg/mL of NQs was added in a solution with PBS buffer (pH 7.4, 100 mL) at a temperature of 25 °C and 37 °C. Then, it was kept under constant magnetic stirring (500 rpm) for 48 h. The in vitro drug release study was followed by ultraviolet-visible spectroscopy using a DU 730 UV-Vis spectrophotometer (λ = 320 nm) [30]. The concentrations of the drug released in the solution were determined at different time intervals, for which aliquots of the volume were extracted as follows: 0, 5, 10, 15 and 30 min. Subsequently, the concentrations were measured every 30 min up to 3 h, and to finish, they were read at 24 and 48 h. A quantity of 1 mL of the solution was extracted for analysis and reintroduced into the PBS buffer medium. Data were plotted and analyzed using Origin (OriginLab Corporation, Northampton, MA, USA) and Excel software (Microsoft 365, Microsoft S. de R.L de C.V, Ciudad de Mexico, Mexico), and the study was performed in triplicate.

### 2.10. Mathematic Model 

To describe the physical mechanism in NQs, mathematical models such as the Weibull (3), Higuchi (4), Hixson–Crowell (5), Korsmeyer–Peppas (6) and Lindner–Lippold (7) models were applied (Table 2).

M = M_0_ [1−e^(−(t − T)b/a)^] (3)

Mt/M∞ = k√t (4)

M_0_^√2^ − M_t_^√2^ = k*_s_*t (5)

Mt/M∞ = (kt)^n^ (6)

Mt/M∞ = k_1_∙t^n^ + b (7)

For this model, the first 3 h of release was used to rule out a burst effect, where, in the Weibull model, **¨b¨** is the value of the power of time, **¨a¨** is the scale parameter defines the time scale of the process, that is, time dependence, and **¨**T**¨** is the location parameter, represents the lag time before the onset of the dissolution or release process, and **¨t¨** is the time of the experiment. For the Korsmeyer–Peppas model, **¨k¨** is a kinetic constant, **¨t¨** is the time of the experiment, and ¨**n**¨ is a diffusional exponent; in the Lindner–Lippold model, **¨b¨** is the representation of the burst effect; in the Higuchi model, **¨k¨** is the kinetic constant, **¨t¨** is time of the experiment; and in the Hixson–Crowell model, **¨k_s_¨** is the kinetic constant.

### 2.11. In-Vitro Assay

Human colorectal cancer cells (Caco-2), hepatocellular carcinoma (HepG2) and human dermal fibroblasts (HDFa) obtained from the American Type Culture Collection (ATCC, Manassas, VA, USA) were cultured in Petri dishes with Dulbecco’s Modified Eagle’s Medium (DMEM) (Gibco©, Grand Island, NY, USA) supplemented with fetal bovine serum (FBS) (Gibco©, Grand Island, NY, USA) 10% and 1% of a commercial mixture of streptomycin and penicillin (Pen Strep Gibco©, Grand Island, NY, USA). The methodology proposed by Ma et. al. [32] was adopted with slight modifications, which are detailed in this section. The cells were incubated at 37 °C in a 5% CO_2_ atmosphere. To evaluate the effects of NBs, NQs, quercetin and inulin on cell viability, the cells were seeded in 96-well plates (concentration of 5 × 10^5^ cells/mL) and incubated for 24 h. All samples were standardized to the quercetin concentration and inulin non-charged nanoparticles (NBs) were used as a negative control; the plates were incubated for 24 h.

Measurements were made with MTS (3-(4,5-dimethylthiazol-2-yl)-5-(3-carboxymethoxyphenyl)-2-(4-sulfophenyl)-2H-tetrazolium) and PMS (phenazine methosulfate) as a stabilizer. The cell viability was measured according to the manufacturer’s instructions of the Cell Titel 96 Aqueous One Solution Kit^®^ (Promega Corporation, Madison, WI, USA). Briefly, 20 uL of Cell Titel 96 Aqueous One Solution Kit^®^ (Promega Corporation, Madison, WI, USA) was added to cells, then they were incubated for 30 min, and finally absorbance was measured at 490 nm on a 96-well plate reader (Synergy HT, Bio-Tek, Winooski, VT, USA). Cell viability was calculated by dividing the absorbance of the treated cells by the absorbance of the control (untreated) cells, and this ratio was expressed as a percentage. The study was performed in triplicate.

### 2.12. Nitric Oxide Assay

Murine macrophage cells (RAW 264.7) were cultured in DMEM-F12 (Gibco©, Grand Island, NY, USA) supplemented with 10% FBS and 1% of the commercial streptomycin-penicillin mixture (Pen Strep Gibco©, Grand Island, NY, USA), and then incubated at 37 °C in a 5% CO_2_ atmosphere (NuAire, Plymouth, MN, USA). Cells were seeded in 96-well plates (5 × 10 ^5^ cells/mL) and allowed to adhere for 24 h. The next day, 50 μL of all samples were added, all samples were standardized to the quercetin concentration, and inulin non-charged nanoparticles (NBs) were used as a negative control. After 4 h, half of the wells were stimulated with lipopolysaccharides (LPS) from *Salmonella enterica* serotype typhimurium (L7261, Sigma-Alrich, St. Louis, MO, USA) at 10 µg/mL (final concentration), while the other half were used as controls for each sample.

The CellTiter 96 Aqueous One Solution kit (Promega, Madison, WI, USA) was used to assess the cell viability following the previously described procedure. Nitric Oxide (NO) production was indirectly measured by means of nitrite determination, using the Griess Reagent System (Promega, Madison, WI, USA). Absorbance measures were performed at 550 nm according to the manufacturer’s instructions using a microplate reader (Synergy HT, Bio-Tek, Winooski, VT, USA). The results were expressed as a percentage of NO production [33], and obtained in triplicate.

### 2.13. Statistical Analysis

The data were expressed as the mean ± SD of three independent experiments and were subjected to statistical ANOVA. A value of *p* < 0.05 was considered significant. GraphPad Prism software 9 (GraphPad software, San Diego, CA, USA) was used for the statistical analysis.

## 3. Results

### 3.1. Encapsulation of Flavonoids by Spray-Drying Method

**Figure 3:**
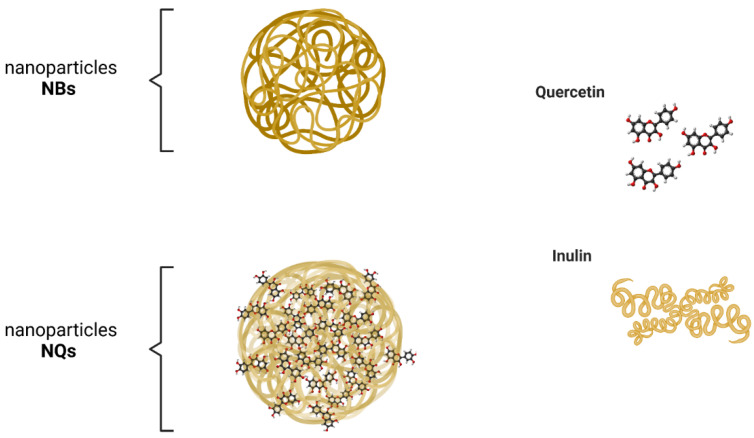
Schematic illustration of synthetized nanoparticles. NBs are the potential structure of unloaded inulin nanoparticles. NQs represents the potential structure of inulin nanoparticles loaded with quercetin by spray drying.

**Figure 4:**
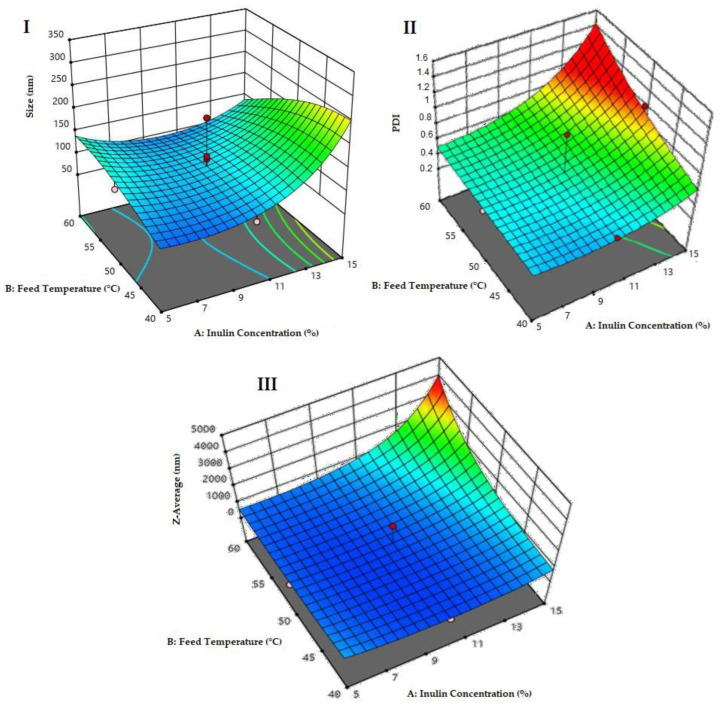
Optimization surface graphs. (**I**) Surface plot for size response (nm), *p*-value of 0.023. (**II**) Surface graph for the analysis of the polydispersity index (PDI) response, with a total *p*-value of 0.683. (**III**) Surface graph for the analysis of the Z-Average response, with a *p*-value of 0.217.

Figure 3 illustrates a potential structure of the nanoparticles loaded with quercetin generated in this study. The experimental design presented in Table 1 was used to optimize the size minimization, PDI and Z-Average, through nanoparticle formulation (inulin concentration %) and conditions of spray-drying synthesis, with a total of 21 experiments (Figure 4). In Figure 4, the PDI and Z-Average surface graphs show a similar behavior, namely that at higher feed temperatures and higher inulin concentrations, the PDI and Z-Average values increased. The inulin concentration had a direct relationship with the size, PDI and Z-Average. The best conditions for the spray-drying process and nanoparticle formulation were 120 °C for the inlet temperature, 60 °C for the outlet temperature, 45 °C for the feed temperature and 10% for the inulin concentration, respectively (Figure 4). As a result of the optimized conditions, two fine powders of loaded (NQs) and unloaded (NBs) nanoparticles were obtained. The performance in the recovery of nanoparticles was 56.8 ± 4.9% and 64.9 ± 3.3% for NBs and NQs, respectively.

### 3.2. Chemical Stability, Zeta Potential and Mean Particle Size Analysis

The zeta potential is the electrostatic potential in the electrical double layer surrounding a nanoparticle in a solution and it can affect the pharmacokinetic properties in the body or may affect the phagocytosis of the nanoparticles in the bloodstream or cell [34]. On the other hand, the PDI is used to describe the degree of non-uniformity of a size distribution of particles; the numerical value of the PDI ranges from 0.0 (for a perfectly uniform sample with respect to the particle size) to 1.0 (for a highly polydisperse sample with multiple particle size populations) [35]. 

**Figure 5:**
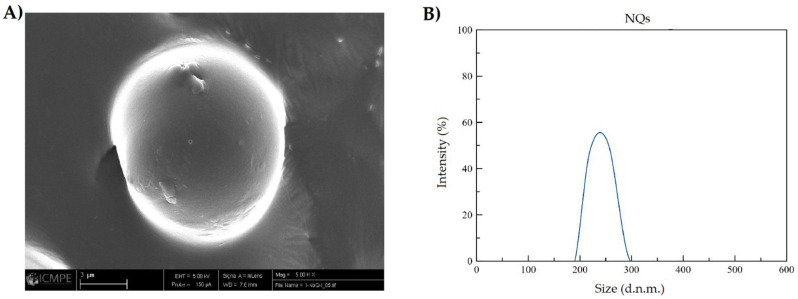
Size characterization of nanoparticles loaded with quercetin. (**A**) SEM image of inulin nanoparticles loaded with quercetin (NQs) (5000× magnification, scale 3 µm). (**B**) DLS NQs reconstituted in organic solvent (absolute ethanol).

**Figure 6:**
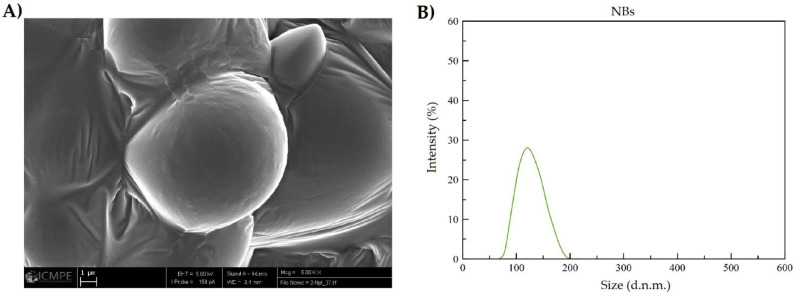
Size characterization of non-charged nanoparticles. (**A**) SEM image of inulin non-charged nanoparticles (NBs) (5000× magnification, scale 1 µm). (**B**) DLS NBs reconstituted in organic solvent (absolute ethanol).

Figure 5B and Figure 6B, show that NQs were higher in size than the NBs, with values of 231.4 ± 10.32 and 118.15 ± 6.57 nm, respectively. There was an increase of 95% in the sizes of NQs compared with NBs. The distribution presented a unimodal trend in both nanoparticles (Figure 5 and Figure 6).

The results of the zeta potential of NQs were significantly different compared to those of NBs. In addition, the medium in which the nanoparticles were dissolved, organic or aqueous solution, influenced the final value of the zeta potential, as shown in Table 3.

### 3.3. Drug Content

**Figure 7:**
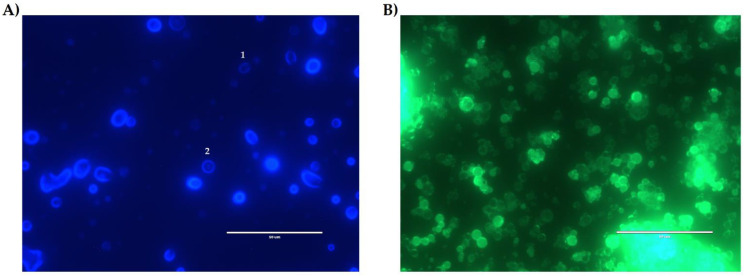
Fluorescence microscopy. (**A**) Inulin nanoparticles loaded with quercetin (NQs) sample with a DAPI filter in organic solution (ethanol). (**B**) NQ sample with GFP filter in organic solution (ethanol). * The numbers in image A indicate: 1. NQs with deficit of loaded quercetin. 2. NQs with quercetin adhered to the surface of the polymeric matrix. White bar represents scale at 50 μm (60× magnification).

The NQs’ encapsulation efficiency was 73.33 ± 7.86%. Less than 30% of quercetin could not be encapsulated. Figure 7A-2 shows that quercetin adhered in the surface in the form of crystals embedded in the surface of the polymeric matrix. This occurred when there was 0.520 ± 0.09 mg/mL of free quercetin in the surface of the NQs.

### 3.4. Thermogravimetric Analysis (TGA)

**Figure 8:**
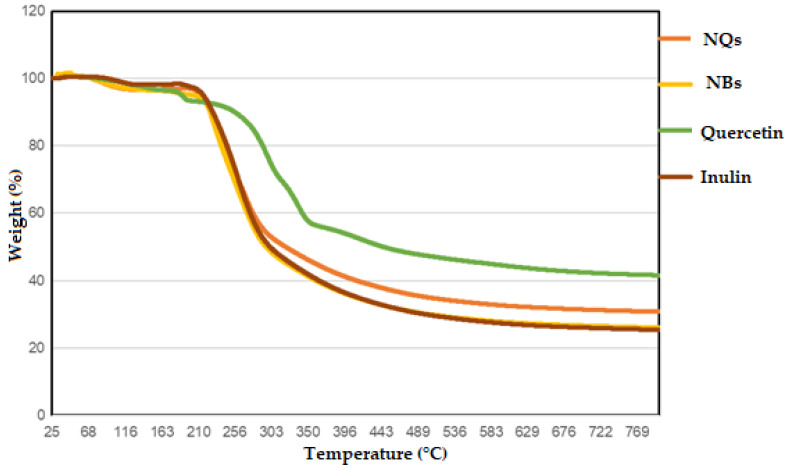
Thermogravimetric analysis (TGA). TGA was measured for quercetin (green line), inulin (brown line), inulin nanoparticles loaded with quercetin (NQs) (orange line) and inulin non-charged nanoparticles (NBs) (yellow line).

The data obtained in the TGA determined the purity and composition of the materials, the drying and ignition temperatures of the materials and the stability temperatures of the samples. In Figure 8, it can be observed that a high temperature (212–216 °C) was needed to decrease the mass of the NQ sample, demonstrating the corrected dryness of the sample. The NQs enhanced the thermal stability at around 1 to 5 °C when 5% of the mass was lost. Taking into account the controls, high temperatures were still needed for quercetin and inulin to observe any decrement in the mass (186–191 °C and 215–218 °C, respectively) (Table 4).

Temperatures needed to be increased until 318–325 °C to reach 50% mass loss for the NQs and needed to be raised to 294–298 °C for the NBs, demonstrating that these nanoparticles are very stable in the presence of high temperatures. The TGA was performed until 800 °C and just 69% mass loss was achieved for the loaded NQs and 75% mass loss was achieved for the NBs. This study demonstrated that both quercetin and inulin are pretty resistant compounds to temperature treatments and that it is possible to make loaded NPs without affecting their thermal properties. 

### 3.5. Powder X-ray Diffraction (XRD)

**Figure 9:**
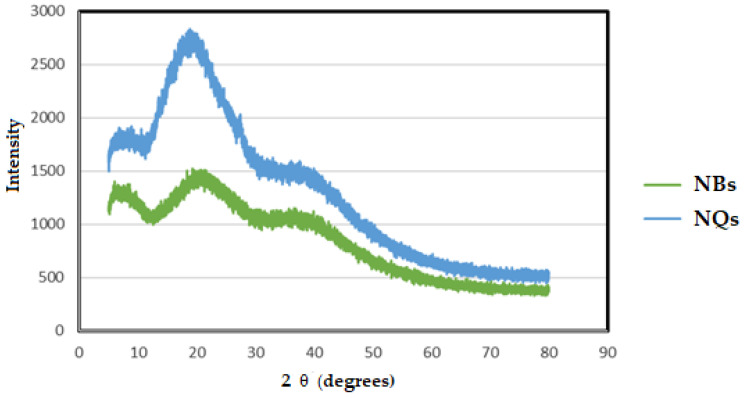
X-ray diffraction (XRD). Pattern of inulin non-charged nanoparticles (NBs) (green line) and inulin nanoparticles loaded with quercetin (NQs) (blue line).

X-ray diffraction (XRD) is a widely used method to analyze a complex formation [36]. Both the NQ and NB samples showed amorphous patterns in the XRD spectra. With a broad peak at around 20 °C, the intensity of the signals increased with the addition of quercetin in the NBs (Figure 9). 

Despite the NBs’ amorphous pattern being similar to the reported inulin pattern, the characteristic crystalline peaks of quercetin, such as 10.2°, 11.0°, 13.6°, 16.6°, 18.0°, 21.9°, 23.4°, 24.5°, 25.3°, 25.8°, 26.2°, 27.1° and 27.8° [36], were not clear.

### 3.6. In-Vitro Drug Release

**Figure 10:**
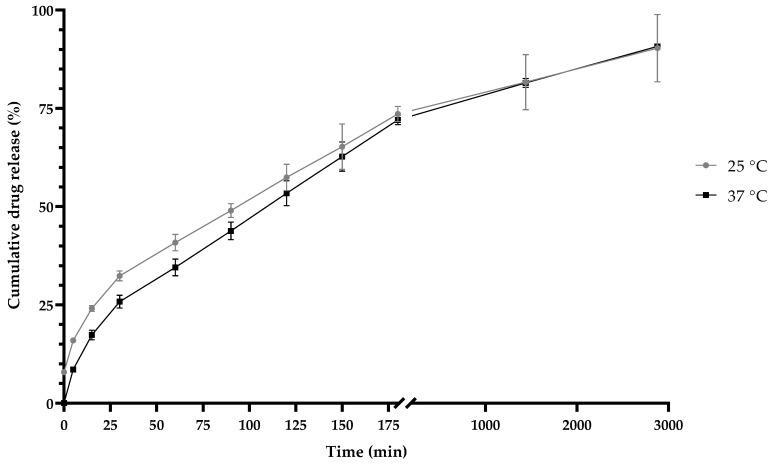
Release kinetics of inulin nanoparticles loaded with quercetin (NQs) at a pH of 7.4 and at different temperatures (25 °C (gray line) and 37 °C (black line)) at 2880 min (48 h).

At both temperatures, 25 °C and 37 °C, at a pH of 7.4, biphasic behavior was observed (Figure 10). The Weibull function (Equation (3)) was used to present the best fit, at both temperatures, with an R^2^ value of 0.99. The value of **“b”** in the Weibull model was 0.6 in the two releases, according to Table 2, and thus, both presented a normal diffusion. According to the **“n”** values in the Korsmeyer–Peppas equation, the two temperature conditions were obtained a sphere with an anomalous type of non-Fickian diffusion. The Lindner–Lippold equation (Table 5) was used to describe more than one type of release in a polymeric matrix, where a burst effect was observed.

### 3.7. Cell Viability

An MTS assay was used to test the cell viability after exposure to the NQ and NB formulations to validate their cytotoxic effects and their potential as chemopreventive treatments. As the nanoparticles were developed for digestive system, aiming at the treatment of colon cancer, it was essential to assess their potential cytotoxicity not only in a Caco-2 model but also in liver cells (HepG2) since, in this organ, 90% of the drugs that enter the body are metabolized.

**Figure 11:**
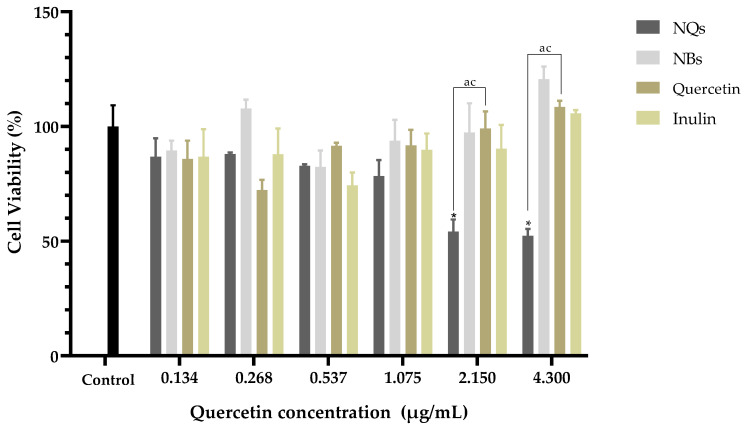
Cell viability in human colorectal cancer cells (Caco-2). Cell viability (%) of inulin nanoparticles loaded with quercetin (NQs), inulin non-charged nanoparticles (NBs), quercetin and inulin at different concentrations (4.3 to 0.134 μg/mL) in human colorectal cancer cells (Caco-2) after 24 h, determined by MTS assay. Values are expressed as mean ± SD. Controls are untreated cells. Significant differences among treatments in the cell lines were determined based on Dunnett multiple comparison test (*p* ≤ 0.05) where * control vs. NQs. Significant differences were determined based on the Student’s t-distribution (*p* ≤ 0.05) where ^ac^ represents NQs vs. quercetin.

Treatment with NQs resulted in a significant decrease in cell viability in contrast to the control cell at concentrations above 2.15 μg/mL (*p* < 0.05) (Figure 11). Free quercetin showed cell viability values above 70% at all concentrations tested. Interestingly, NQs induced a significant reduction in cell viability at concentrations above 0.537 μg/mL compared to free quercetin. In this sense, cells treated with NQs showed a reduction in the viability values with the increasing of the concentration, exhibiting a dose-dependent tendency. As expected, pure free inulin did not produce any relevant cytotoxic effect on the Caco-2 cell line, with the viability remaining above 80% at all concentrations tested. 

**Figure 12:**
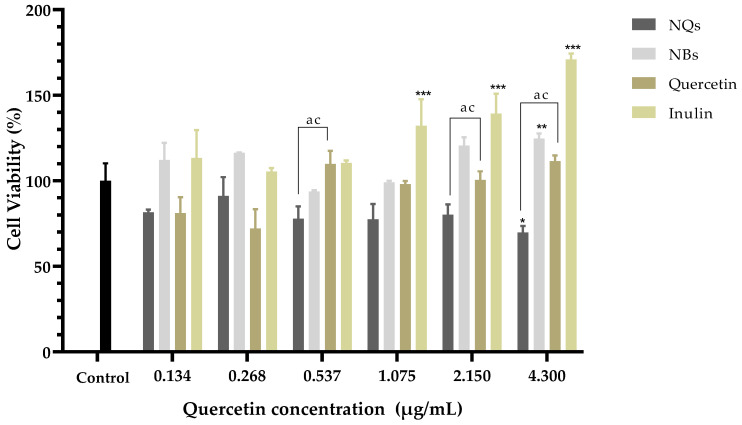
Cell viability in hepatocellular carcinoma (HepG2). Cell viability (%) of inulin nanoparticles loaded with quercetin (NQs), inulin non-charged nanoparticles (NBs), quercetin and inulin at different concentrations (4.3 to 0.134 μg/mL) in hepatocellular carcinoma (HepG2) after 24 h determined using the MTS assay. Values are expressed as mean ± SD. Controls are untreated cells. Significant differences among treatments in the cell lines were determined based on Dunnett multiple comparison test (*p* ≤ 0.05) where * represents control vs. NQs, ** represents control vs. quercetin and *** represents control vs. inulin. Significant differences were determined based on the student’s t-distribution (*p* ≤ 0.05) where ^ac^ represents NQs vs. quercetin.

In HepG2, a human liver cell line, cell viability was examined using the MTS test and was determined after 24 h of incubation of cells with NQs, NBs, quercetin and inulin. As shown in Figure 12, NQs showed a cell viability of around 70% in the highest concentration (4.3 μg/mL). NBs and free inulin did not show a toxic effect; conversely, inulin shown a proliferation of cells at 4.3 μg/mL. This suggests that the nanoparticle matrix, inulin, does not have a cytotoxic effect on HepG2 cells. Free quercetin showed an inhibition of cell viability of around 30% at 0.268 μg/mL compared to NQs, with 10% inhibition at the same concentration. However, at concentrations above 0.537 μg/mL, the cell viability of cells treated with NQs decreased significantly more than that of cells treated with free quercetin.

**Figure 13:**
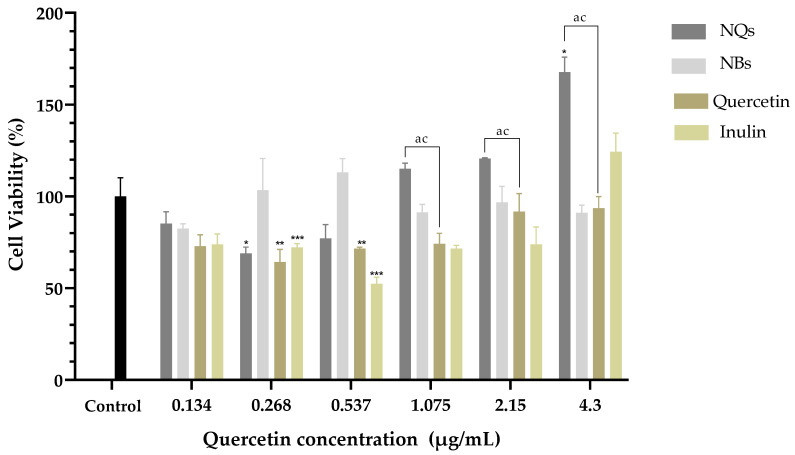
Cell viability in human dermal fibroblast cell line (HDFa). Cell viability (%) of inulin nanoparticles loaded with quercetin (NQs), inulin non-charged nanoparticles (NBs), quercetin and inulin at different concentrations (4.3 to 0.134 μg/mL) in human dermal fibroblast cell line (HDFa) after 24 h, determined using the MTS assay. Values are expressed as mean ± SD. Controls are untreated cells. Significant differences among treatments in the cell lines were determined based on Dunnett multiple comparison test (*p* ≤ 0.05) where * represents control vs. NQs, ** represents control vs. quercetin and *** represents control vs. inulin. Significant differences were determined based on the Student’s t-distribution (*p* ≤ 0.05) where ^ac^ represents NQs vs. quercetin.

HDFa is a human dermal fibroblast cell line; this was examined by the MTS test and was determined after 24 h of incubation of cells with NQs, NBs, quercetin and inulin. As shown in Figure 13, NQs showed greater than 70% cell viability, on average, at all tested doses (0.268–4.3 μg/mL). In contrast, quercetin showed a significant difference with NQs at concentrations of 1.075 to 4.3 μg/mL, where HDFA cells treated with NQs showed higher viability than HDFa cells tested with quercetin. On the other hand, NBs and free inulin did not show a toxic effect with around 80% and 70% cell viability, respectively, at all concentrations.

### 3.8. Nitric Oxide Assay

**Figure 14:**
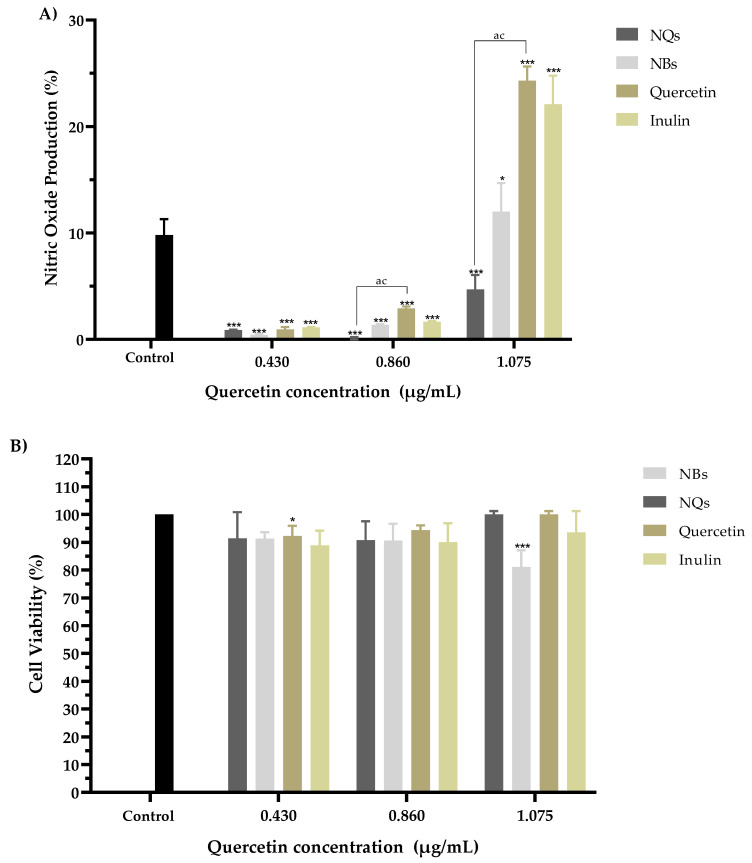
The nitric oxide production assay. Nitric oxide production (%) action of inulin nanoparticles loaded with quercetin (NQs), inulin non-charged nanoparticles (NBs), quercetin and inulin on NO production in Murine macrophage cells (RAW 264.7). (**A**) Effects with 0.430, 0.860 and 1.075 µg/mL quercetin concentrations (all samples were standardized to the quercetin concentration) for 8 h. (**B**) Cell viability of the extracts at 8 h in RAW 264.7 cells at 0.430, 0.860 and 1.075 µg/mL quercetin concentrations (all samples were standardized to the quercetin concentration), with 10 µg/mL of LPS. Each treatment was carried out in triplicate. Evaluations were made using the two-way ANOVA, with Dunnett’s multiple comparisons, where * represents *p* < 0.05, and *** represents *p* < 0.001 compared to the control group. Significant differences were determined based on the Student’s t-distribution (*p* ≤ 0.05) where ^ac^ represents NQs vs. quercetin.

The potential immunomodulatory effects of NQs, NBs, quercetin and inulin were analyzed by measuring the amount of nitric oxide (NO) released. Figure 14A shows the NO activity induced by the samples at concentrations of 0.430, 0.860 and 1.075 μg/mL; the activity is expressed as the % production of nitrites released in RAW 264.7 cells.

A significant decrease in nitric oxide production was observed in all samples compared to the untreated control. A viability assay was performed on the same cells for all four models to rule out any potential for cytotoxicity. It was observed that the decrease in nitric oxide production did not show a toxic effect in RAW 264.7 cells in the concentration range of 0.430 to 1.075 μg/mL in comparison with the control cells without treatment (Figure 14B). 

## 4. Discussion

In this work, the preparation and characterization of NQs formed from an oligosaccharide matrix, inulin, by spray drying, as possible nanoparticles to be administered orally to prevent NCDs such as metabolic syndrome, was described. The use of spray-drying equipment presented a functional tool for the formulation of nanoparticles based on inulin and quercetin. For their optimization, it was observed that the concentration of inulin showed a direct proportionality with respect to the nanoparticle size and PDI (Figure 4). The initial concentration of inulin plays an essential role because the greater the quantity of material that needs to be dried, the greater the viscosity of the solution, which leads to a longer time in the atomizing chamber of the drying equipment, making it difficult for droplet formation to occur and possibly causing the nozzle to clog [37]. Therefore, at a higher concentration of inulin and a higher feeding temperature, inulin tends to present an effect of increasing the polydispersity and Z-Average, due to the formation of agglomerates owing to the properties that produce products that are rich in sugars. They are subjected to conditions that exceed their glass transition point [28]. 

The results obtained by DLS showed an increase in size of 95% between NBs and NQs (Figure 5B and Figure 6B), which confirms the encapsulation of quercetin. The size distribution for NQs was 220–240 nm, which was comparable with the results obtained by Charoenwongpaiboon et al. [38], where the sizes were dependent on the temperature and pH, with dimensions between 95.9 and 115.7 nm for temperatures between 50 and 40 °C. Jiménez-Rodriguez et al. [39] mentioned that molecular weight plays a vital role in the self-assembly of nanoparticles; they noted that the lower the molecular weight, the smaller the nanoparticles obtained. This effect is possible due to the length of their branching; in Figure 5B and Figure 6B, taken by SEM microscopy, it can be seen the NQs and NBs were spherical and had slightly rough surfaces. The images show areas with a high degree of agglomeration. This presence of aggregations and variation in the load of the active principle was influenced by: the collision between particles in the spray-dried equipment, either in the drying zone or in the cyclone zone, or the effect of elevated temperatures on the inulin, which originated from an adhesion between particles of varied sizes [40,41]. Furthermore, in Table 3, it is shown that the sizes of NQs and NBs were influenced by the medium in which the nanoparticles were suspended; in an aqueous medium, a slight relaxation of inulin was reported, with pores generated in the matrix and a swelling of the sphere, due to the relaxation of the polymeric chains. This normally occurs in nanoparticles with inulin form a hydrophilic layer [42].

The zeta potential in the NQ nanoparticles indicated colloidal particle stability in a controlled setting [43]. NQs showed a zeta potential of −13.28 ± 4.3 mV in an organic solvent and −0.178 ± 0.06 mV in H_2_O. In aqueous medium, NQs were considered neutral, because there was zeta potential between −10 and +10 mV. In contrast, in an organic solvent, NQs were less than −30 mV, and thus, these nanoparticles were considered strongly anionic. Since most cell membranes are negatively charged, the value of the zeta potential can affect a nanoparticle’s tendency to permeate membranes [34]. In addition, the results showed an adverse effect for NQs due to hydroxyl groups in the inulin wall material [44] or to the hydrogen bond with water and the deprotonation of the carboxymethyl groups it possesses [45]. The negative zeta potential value (Table 3) suggests a tendency towards aggregation between particles. Jiménez-Sanchez et al. [46] obtained a value of −10 ± 1.8 mV for nanoparticles of inulin. Despite the negative presence in the polymeric wall, no significant aggregation was observed in NQs in an organic medium. Peng et al. [47] mentioned that the presence of another inulin-based compound in nanoparticles can affect the neutral charge of inulin. They observed that nanoparticles with an inulin wall formed strong chains, which caused a steric repulsion between particles. Considering the results of the positive zeta potential of NBs, we can suggest that the presence of quercetin in the formulation modified the neutral charge of inulin. In addition, the surface of the nanoparticles must be kept neutral since a neutral charge attracts the least amount of serum proteins. However, the negative charges of the nanoparticles could induce opsonization and, thus, MPS uptake, but at a reduced rate relative to a positively charged surface—the lesser the opsonization, the greater the efficiency of accumulation in tumors [23]. 

Overall, PDI values of between 0.1 and 0.3 indicated a narrow size particle distribution. Though the PDI values of NQs were slightly above the prescribed limits for narrow size distributions, they cannot be considered as completely heterogenous [48]. Table 3 shows different PDI values between NBs and NQs, and that quercetin loading significantly increased the polydispersity index in NQs.

The fluorescence microscope and UV-vis measurements confirmed that inulin should be limited relative to quercetin because there was the presence of free quercetin on the surface of the nanoparticles. As for the EE% results (Figure 7), the percentage observed in NQs was 73.33 ± 7.86%. The characteristics and EE% of NQs are similar to those reported by Morelo et al. [40]; they encapsulated flavonoids (quercetin and epicatechin) in an inulin matrix by spray drying. They obtained a yield of 78.1 ± 1.0% with a feed temperature of 90 °C and an inlet temperature of 160 °C. In additon, this treatment presented surface irregularities and agglomeration, which were similar to those observed in SEM (Figure 5A and Figure 6A).

The high thermal stability of inulin and quercetin has been reported. For example, Wahbi [49], analyzed the thermal behavior of an inulin extract that started to decompose at ≈235 °C [49], and of raw inulin at 215 °C [50]. On the other hand, the reported thermal degradation of quercetin was observed between 245 and 390 °C [36], which is similar to the results obtained in the present project (Figure 8). The observed higher temperatures range of NQs could be due to the physical and chemical interactions created between both inulin and quercetin [49]. No thermal data were found in the literature to directly compare the thermal behavior of NBs and NQs.

NQs and NBs presented an amorphous XRD pattern that was similar to inulin, and these patterns coincided well with previous studies [51,52]. Although quercetin is reported to have a crystalline structure, its addition to the NBs improves the amorphous phase by incrementing the intensity of the signals, with this being due to the chemical interaction between both compounds [36].

The drug release profiles of NQs in a dissolution medium, at a pH of 7.4, at two different temperatures (25 °C and 37 °C) showed the same behavior. In the first phase (0 to 30 min), it was attributed to the flavonoid present in the superficial part of the matrix—this phase is known as the explosion phase. In the second phase, a sustained release phase was observed, where the release was constant after 60 min (Figure 7) [26]. We obtained a sphere with an anomalous non-Fickian type of diffusion according to values in Table 4. This type of mechanism was governed by the diffusion and swelling of the polymeric matrix. This chain rearrangement and diffusion process created anomalous time-dependent diffusion effects.

Figure 11 shows that the Caco-2 cell viability was significantly lower in cells treated with NQ nanoparticles (52.27 ± 3.0%) compared to cells treated with quercetin (108.50 ± 2.70%) at 4.3 μg/mL. On the other hand, NBs and inulin did not significantly affect the cell viability. NQs exhibited stronger inhibitory effects at concentrations above 1.075 μg/mL. The results suggested that NQ nanoparticles promoted the internalization of quercetin in Caco-2 cells, contributing to the better anti-proliferative activities. Therefore, incorporating inulin in the nanoparticles did not affect the cell integrity, indicating that the formulations have biocompatibility. In HepG2 cells at a 4.3 μg/mL concentration, the NQs presented a decreased cell viability of around 30%. At 0.268 μg/mL, free quercetin exhibited more significant cytotoxicity than NQ, but at a concentration of 0.537 μg/mL, the behavior was the opposite—a more substantial decrease in cell viability was observed for NQ than for quercetin. The result could be a result of NQs promoting the internalization of Q, thereby contributing to the better anti-proliferative activities. Li et. al. [53] reported that nanoparticles based on *Hohenbuehelia serotina* polysaccharides (HSP), used for the delivery of quercetin, presented significant anti-proliferative activities against cervical carcinoma cells (HeLa) in dose-dependent manners. In addition, in comparison with free quercetin (IC_50_ value of 271.3 μg/mL), their nanoparticles exhibited a stronger inhibitory effect on HeLa cells, with the IC_50_ value of 124.5 μg/mL, suggesting that the anti-proliferative activity of quercetin was significantly enhanced by encapsulation with HSP.

In both cell lines, Caco-2 and HepG2, it was observed that no cytotoxicity was observed at concentrations of 0.134–4.3 μg/mL with regard to NBs (without quercetin nanoparticles), and the cell viability remained above 85% in all treatments. Jiménez-Sánchez et al. [46] analyzed different formulations of nanoparticles based on polysaccharides and found that they did not show cytotoxicity in peripheral blood mononuclear cells (PBMC’s). Thus, it was suggested that they are safe in their administration as a vehicle, and due to their versatility and low toxicity, they have been used as a vehicle for the release of chemotherapeutic agents. Nevertheless, in nontumoral cell lines such as human dermal fibroblast (HDFa), NQs showed a proliferative effect at the highest concentrations (1.075 to 4.3 μg/mL). This suggests that NQs have significant cytotoxicity effects against Caco-2 and HepG2 cells and minimal effects against normal HDFa cells.

Figure 14 shows the synergistic effect of inulin and quercetin on NQs at a concentration of 1.075 μg/mL. This difference in the percentage of NO production could be due to two factors: the size and synergy between the matrix and the active compound. The size of the NQs presents an advantage for the internalization of the molecule into the cell, according to Garrido-Cano et al. [54]. The authors reported that 179-nm-sized silica nanoparticles with hyaluronic acid achieved more efficient internalization in two cell lines (MDA-MB-231 and MCF10A), as compared to nanoparticles with sizes above 250 nm. [55]. The NQs had a size of 231.4 ± 10.34 nm in organic solution, which was within the range of sizes described by Garrido-Cano and colleagues. The results indicated that quercetin that was not encapsulated in the NQs nanoparticles was internalized and had a more significant effect on cell viability than free quercetin. Inulin and quercetin, on the other hand, functioned as inhibitors of the same inflammation signaling pathways and regulators of the proinflammatory effect [54,56,57].

## 5. Conclusions

A CCFCD design experiment made it possible to optimize the spray-drying conditions and synthesis formulation of nanoparticles of inulin, which were used as carriers and loaded with quercetin. The optimized formulation obtained a size of 231.4 ± 10.32 nm in organic solvent and 289.75 ± 16.3 nm in water. Nanoparticles of smooth and regular shape with a zeta potential of −13.28 ± 4.3 mV were obtained in an organic solvent, and nanoparticles of −0.178 ± 0.06 mV were obtained in water. The in vitro release of quercetin in NQs improved when it was in a basic medium at a temperature of 25 °C and 37 °C, and more than 70% of the active ingredient could be released after 3 h of administration. Quercetin’s release was governed at least two processes, namely diffusion and relaxation of the spherical nanoparticles’ polymeric chains, via a non-Fickian release mechanism.

The MTS assay revealed significant cytotoxicity of NQs up to a concentration of 4.3 μg/mL in Caco-2, and the internalization of NQs led to the potential toxic effect of quercetin. In HepG2 cells, the NQs, at all concentrations, showed a cell viability of more than 70%. In the case of HDFa cells, the NQs showed a minimal inhibition effect at small concentrations; on the contrary, at high concentrations, a proliferative effect was observed. Overall, the results of the experiments suggest that the developed NQs constitute a promising and safe colon-targeted drug delivery system possessing good biocompatibility and stability. Furthermore, the combination of spray drying and biopolymers such as inulin for the creation of nanoparticles for the release of bioactive compounds such as quercetin allows the creation of more cost-effective systems and, therefore, facilitates the use of nanoparticles in a variety of applications. Further research should be carried out to assess digestive resistance based on in vitro digestion and associated transport mechanisms/internalization pathways, as well as to test its performance and efficacy in vivo in an animal model.

## Figures and Tables

**Table 1:** Experimental face-centered central composite design (CCD). Independent variables and levels.

Variable	Low Point	Central Point	High Point
Inulin concentration (%)	5	10	15
Feed temp. (°C)	40	50	60
Temp. in (°C)	100	150	200
Temp. out (°C)	60	80	100

**Table 2:** Release exponent (n) in the Korsmeyer–Peppas and drug transport mechanisms [31].

Thin Film	Cylindrical Sample	Spherical Sample	Drug Release Mechanism
0.5	0.45	0.43	Fickian diffusion
0.5 < n < 1.0	0.45 < n < 0.89	0.43 < n < 0.85	Anomalous (non-Fickian transport)
1	0.89	0.85	Type II transport

**Table 3:** Zeta potential, polydispersity index (PDI) and size of inulin nanoparticles loaded with quercetin (NQs) and non-charged nanoparticles (NBs) at 37 °C in two different mediums.

	Organic Phase (Ethanol)			Aqueous Phase (H_2_O)		
	Zeta Potential (mV)	Size (nm)	PDI	Zeta Potential (mV)	Size (nm)	PDI
**NQs**	−13.28 ± 4.3	231.4 ± 10.32	0.464 ± 0.12	−0.178 ± 0.06	289.75 ± 16.3	0.94 ± 0.08
**NBs**	1.4 ± 1.9	118.15 ± 6.57	0.243 ± 0.36	−11.09 ± 1.5	102.68 ± 12.8	0.201 ± 0.1

**Table 4:** TGA data, % of weight loss vs. increment of temperature in loaded and unloaded nanoparticles, inulin and quercetin.

Samples	% of Mass Loss		
	Onset Degradation Temperature (Ti) 5%	Critical Degradation Temperature 50%	25–42%
**NQs**	212–216 °C	318–325 °C	31% (688–797 °C)
**NBs**	190–211 °C	294–298 °C	25% (819–827 °C)
**Quercetin**	186–191 °C	299–304 °C	40% (887–897 °C)
**Inulin**	215–218 °C	439–453 °C	25% (770–897 °C)

NQs: inulin nanoparticles loaded with quercetin. NBs: inulin non-charged nanoparticles.

**Table 5:** Mathematical models used for the prediction of kinetic release in the first 3 h.

Mathematical Model	Temperature							
	25 °C				37 °C			
**Higuchi**	**k**		**R^2^**		**k**		**R^2^**	
	4.924		0.988		5.264		0.289	
**Weibull**	**b**		**R^2^**		**b**		**R^2^**	
	0.602		0.990		0.621		0.993	
**Hixson–Crowell**	**k**		**R^2^**		**k**		**R^2^**	
	0.008		0.986		0.008		0.989	
**Korsmeyer–Peppas**	**k**	**n**		**R^2^**	**k**	**n**		**R^2^**
	3.077	0.601		0.995	5.862	0.481		0.975
**Lindner–Lippold**	**b**	**n**		**R^2^**	**b**	**n**		**R^2^**
	7.727	0.601		0.991	1.086	0.621		0.994

## Data Availability

The data that support the findings of this study are available from the corresponding author, R.A.C.-S., upon request.
